# Affected cortico-striatal-cerebellar network in schizophrenia with catatonia revealed by magnetic resonance imaging: indications for electroconvulsive therapy and repetitive transcranial magnetic stimulation

**DOI:** 10.1093/psyrad/kkad019

**Published:** 2023-10-19

**Authors:** Xiao-Fan Liu, Shu-Wan Zhao, Zachary Kratochvil, Jia-Cheng Jiang, Di Cui, Lu Wang, Jing-Wen Fan, Yue-Wen Gu, Hong Yin, Jin-Jin Cui, Xiao Chang, Long-Biao Cui

**Affiliations:** Department of Radiology, Xi'an Gaoxin Hospital, Xi'an 710075, China; Schizophrenia Imaging Lab, Fourth Military Medical University, Xi'an 710032, China; Schizophrenia Imaging Lab, Fourth Military Medical University, Xi'an 710032, China; Composable Analytics, Cambridge, MA 02140, USA; Department of Radiology, The Second Medical Center, Chinese PLA General Hospital, Beijing 100853, China; Schizophrenia Imaging Lab, Fourth Military Medical University, Xi'an 710032, China; Schizophrenia Imaging Lab, Fourth Military Medical University, Xi'an 710032, China; Schizophrenia Imaging Lab, Fourth Military Medical University, Xi'an 710032, China; Schizophrenia Imaging Lab, Fourth Military Medical University, Xi'an 710032, China; Department of Radiology, Xi'an People's Hospital, Xi'an 710004, China; Department of Radiology, The Second Medical Center, Chinese PLA General Hospital, Beijing 100853, China; Institute of Science and Technology for Brain Inspired Intelligence, Fudan University, Shanghai 200433, China; Schizophrenia Imaging Lab, Fourth Military Medical University, Xi'an 710032, China; Department of Radiology, The Second Medical Center, Chinese PLA General Hospital, Beijing 100853, China; Shaanxi Provincial Key Laboratory of Clinic Genetics, Fourth Military Medical University, Xi'an 710032, China; Department of Radiology, The First Affiliated Hospital of Xi’an Jiaotong University, Xi'an 710061, China

**Keywords:** catatonia, schizophrenia, magnetic resonance imaging, neurostimulation

## Abstract

Catatonia is a psychomotor syndrome that can occur in a broad spectrum of brain disorders, including schizophrenia. Current findings suggest that the neurobiological process underlying catatonia symptoms in schizophrenia is poorly understood. However, emerging neuroimaging studies in catatonia patients have indicated that a disruption in anatomical connectivity of the cortico-striatal-cerebellar system is part of the neurobiology of catatonia, which could serve as a target of neurostimulation such as electroconvulsive therapy and repetitive transcranial magnetic stimulation.

## Introduction

**Figure 1:**
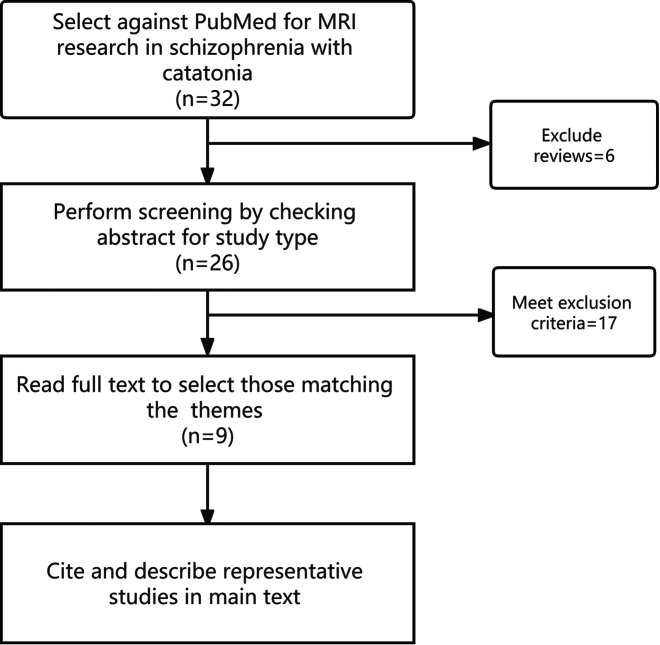
Strategy of literature review in this study. Six reviews were excluded at this screening stage. After full-text reading, 17 papers were excluded because they did not meet the inclusion criteria. Nine recent reports were included to review the MRI findings in schizophrenia with catatonia.

Catatonia is a striking psychomotor syndrome characterized by varying motor, affective, and behavioral symptoms. The clinical manifestations of the syndrome include reduced responsiveness (catalepsy, stupor, mutism), repetitive movements and behaviors (stereotypy, echolalia, echopraxia, mannerisms), bizarre postures (waxy flexibility, posturing), and intense anxiety or agitation (Fink and Taylor, 2009; Organization, 2013; Tandon *et al*., 2013). Catatonia has been found to occur in as many as 7-31% of psychiatric inpatients (Daniels, 2009; Lee *et al*., 2000; Rosebush *et al*., 1990; Rosebush and Mazurek, 2010; Stuivenga and Morrens, 2014; Taylor and Fink, 2003) and is associated with high mortality if left untreated (Cornic *et al*., 2009; Tuerlings *et al*., 2010). Currently, the treatment of catatonia includes medication and physical therapy, such as electroconvulsive therapy (ECT) or repetitive transcranial magnetic stimulation (rTMS). ECT is currently the common treatment for catatonia. Even though catatonia has been known >140 years ago, the exact neural mechanism behind the disease remains unclear. Catatonia is most commonly associated with affective disorders and schizophrenia (Chalasani *et al*., 2005; Morrison, 1975; Tuerlings *et al*., 2010) but also occurs in neurologic and other medical conditions (Ahuja, 2000; Gelenberg, 1976; Saddawi-Konefka *et al*., 2014). Irrespective of the underlying illness, catatonia patients exhibit marked similarities in clinical symptoms and treatment response (Smith *et al*., 2012; Tandon *et al*., 2013), suggesting that there may be a common neurobiological mechanism. To date, a limited number of neuroimaging studies have attempted to unravel the neurobiology of catatonia, mainly using functional magnetic resonance imaging (fMRI). These studies show abnormalities of orbitofrontal, (medial) prefrontal (Northoff *et al*., 2004; Richter *et al*., 2010), and parietal regions (Northoff *et al*., 1999, 2000; Satoh *et al*., 1993), as well as the premotor cortex (Northoff *et al*., 2004, 1999; Richter *et al*., 2010), and supplementary motor area (SMA) (Scheuerecker *et al*., 2009; Walther *et al*., 2017a) in catatonia patients compared to healthy controls and schizophrenia without catatonia. If the results matter, how this collective of implicated brain regions contributes to the manifestation of catatonia on the behavioral level remains to be determined. Here, we introduce the research progress of MRI changes of brain structure and function in schizophrenia with catatonia and their clinical implications for neurostimulation, i.e., ECT to rTMS, which could provide a reference for the clinical treatment of schizophrenia with catatonia. In the following sections, we summarize studies on schizophrenia with catatonia. This paper used PubMed to retrieve relevant literature. The retrieved documents included original research and review articles, and the search string was set as "Schizophrenia and Catalonia and Magnetic Resonance Imaging." Six reviews were excluded according to the title and abstract. After full-text reading, 17 papers were excluded because they did not meet the inclusion criteria. Nine recent reports (listed in Table 1) were included in the article. The literature search strategy for this review is summarized in Fig. 1. To focus on the pathology with homogeneity, we only discussed catatonia in schizophrenia.

**Table 1:** Examples of studies on schizophrenia with catatonia.

Sample and data	Findings in schizophrenia with catatonia
Northoff *et al*.[Northoff *et al*., 2000]: catatonia patients (*n* = 10); healthy controls (*n* = 10)	Both emotional and motor symptoms in catatonia are closely related to left sensorimotor and right parietal alterations.
Scheuerecker *et al*.[Scheuerecker *et al*., 2009]: schizophrenia patients (*n* = 12); healthy controls (*n* = 12)	During the self-initiated movements, patients showed less activation in the SMA and the prefrontal and parietal cortices.
Walther *et al*.[Walther *et al*., 2017a]: schizophrenia patients (*n* = 42); healthy controls (*n* = 41)	SMA resting-state perfusion may surve as a marker of current catatonia in schizophrenia.
Walther *et al*.[Walther *et al*., 2017b]: schizophrenia patients (*n* = 46); healthy controls (*n* = 44)	Thalamocortical hyperconnectivity was associated with motor abnormalities in patients.
Fritze *et al*.[Fritze *et al*., 2020]: 111 SSD patients	In catatonic patients, significant correlations were detected between NCRS motor scores and the whole brainstem.
Hirjak *et al*.[Hirjak *et al*., 2019]: 56 SSD patients	Distinct dimensions of catatonia are associated with different patterns of abnormal brain structure.
Hirjak *et al*.[Hirjak *et al*., 2020]: 87 SSD patients	NCRS behavioral scores were associated with a joint structural and functional system that predominantly included cerebellar and prefrontal/cortical motor regions.
Wasserthal *et al*.[Wasserthal *et al*., 2020]: 111 SSD patients and 28 healthy controls	Structural reorganization of WM bundles connecting orbitofrontal/parietal, thalamic, and striatal regions contributed to catatonia in SSD patients.
Dean *et al*.[Dean *et al*., 2020]: SSD patients with catatonia (*n* = 43); SSD patients without catatonia (*n* = 43); healthy controls (*n* = 86).	Psychomotor cognitive functioning may differentiate psychosis patients with catatonia from those without catatonia.

## MRI Findings in Schizophrenia with Catatonia

One part of the network found to be affected in catatonia patients is the bilateral medial orbitofrontal cortex. This functionally complex region has been implicated in emotional appraisal and regulation via its link to limbic regions (Etkin *et al*., 2011). The medial orbitofrontal cortex mediates behavioral inhibition in negative emotions (Goldstein *et al*., 2007; Silbersweig *et al*., 2007). A previous study found decreased structural connectivity between the bilateral medial orbitofrontal cortex and striatum in catatonia patients, which could cause impaired emotion regulation of anxiety and contribute to reduced responsiveness of patients (Northoff *et al*., 2004; Richter *et al*., 2010). Future studies in larger samples of catatonia patients or using more sensitive measurements of anxiety may help elucidate the contribution of the orbitofrontal cortex to catatonia symptoms.

Previous studies have suggested that increased radial diffusivity along fiber tracts is likely related to de- or dysmyelination rather than axonal pathology, which is more likely to be reflected in axial diffusivity measurements (Budde *et al*., 2009; Harsan *et al*., 2006; Klawiter *et al*., 2011; Song *et al*., 2003, 2002). However, factors such as axonal density and crossing fibers also influence radial diffusivity (Wheeler-Kingshott and Cercignani, 2009), and these factors are difficult to disentangle from myelination properties with current *in vivo* imaging techniques. Therefore, we tentatively suggest that catatonia may involve changes in the myelination of corticostriatal connections. Some of the previous neuroimaging studies in catatonia demonstrated functional abnormalities in these region (Northoff, 2002), including alterations in the cortico-striatal functional network connectivity and abnormalities in the SMA during rest (Walther *et al*., 2017a). Other studies implicated the premotor cortex in catatonia (Northoff *et al*., 1999; Richter *et al*., 2010). Walther *et al*. reported that the catatonia factor correlated with functional connectivity between the left thalamus and the bilateral primary motor cortex (Walther *et al*., 2017b). It is also said that higher connectivity is associated with severe catatonic symptoms. In addition, Walther *et al*. reported the severity of catatonia was correlated with the hyperperfusion of the SMA (Walther *et al*., 2017a).

Some key cortical motor areas below the skull may be the entry node for non-invasive brain regulation. Therefore, rTMS may help improve the dysfunctional motor network of patients with schizophrenia. Currently, most of the targets regarding rTMS for the treatment of catatonia are located in the dorsolateral prefrontal cortex (DLPFC) (Sharma *et al*., 2018; Stip *et al*., 2017; Trojak *et al*., 2014).

The striatal regions implicated in previous studies, including the nucleus accumbens, caudate nucleus, and putamen, are involved in a wide range of volitional, motor, and emotional aspects of human behavior (Alexander and Crutcher, 1990; Cummings, 1993). The striatum is an essential regulator of motor control, particularly in terms of initiation (Cunnington *et al*., 2002; Hauber, 1998) and inhibition (Chevrier *et al*., 2007; Frank *et al*., 2001; Verbruggen and Logan, 2008; Vink *et al*., 2005) of motor response. Reduced output from the striatum to the frontal cortex may lead to difficulties in initiating or terminating ongoing movements, which could underlie symptoms such as akinesia, posturing, stereotypic movements, and echolalia (Jahanshahi *et al*., 2015; Northoff, 2002). Indeed, patients with idiopathic basal ganglia calcification, an inherited neurological disorder, also exhibit catatonia-like symptoms (Brunoni *et al*., 2009; Ishitobi *et al*., 2014; Saito *et al*., 2010), and blockade of striatal dopamine receptors has been shown to cause catalepsy in animal studies (Hauber *et al*., 2001). In all, there is strong evidence to suggest a central role for the basal ganglia in the pathophysiology of catatonia. Extending these findings means that disruptions in the anatomical connections linking basal ganglia to the cortical regions contribute to the development of catatonia.

Schizophrenia spectrum disorders (SSD) include a variety of diseases, most of which are schizophrenia. A recent study examined brainstem volume in catatonic SSD patients, and a significant correlation between the NCRS (Northoff Catatonia Rating Scale) motor score and the whole brainstem was found. It suggests abnormal total brainstem volume is essential in catatonia (Fritze *et al*., 2020). Furthermore, Hirjak *et al*. (2019) argued that cortical changes in frontoparietal regions might drive the catatonic SSD. Studies have shown that orbital-frontal/parietal lobe, thalamus, and striatal structural abnormalities are mainly present in SSD patients with catatonia compared with SSD patients without catatonia (Hirjak *et al*., 2020; Wasserthal *et al*., 2020). However, Dean *et al*. (2020) showed that there is no difference in brain structure in SSD patients with catatonia and those without catatonia. However, compared with healthy controls, the patient groups had reduced gray volume in several areas, including insula, anterior cingulate, medial frontal, and temporal cortices. In addition, SSD patients with catatonia had more significant cognitive difficulties than SSD patients without catatonia (Dean *et al*., 2020). Compared to SSD patients without catatonia, patients with catatonia had increased static functional connectivity in the cerebellar networks and decreased low-frequency oscillations in the basal ganglia networks (Sambataro *et al*., 2021).

## Implications of MRI Findings in Schizophrenia with Catatonia for ECT and rTMS

Although some literature describes the improvement of catatonic symptoms after ECT, there is also negative findings. The case report showed a patient failed to improve after prolonged ECT and TMS treatment (Stip *et al*., 2017). In recent years, rTMS has been considered a new option for treating neuropsychiatric diseases (Wu *et al*., 2021). rTMS may be a safe alternative treatment strategy for patients who failed multiple drug interventions and had safety problems with ECT. To date, only a few reports on TMS use in catatonia have been published. Although TMS appears promising, conclusions can only be drawn with more studies or a more significant number of case reports.

In line with an individualized strategy for MRI-guided and navigated neurostimulation in schizophrenia (Wu *et al*., 2021), MRI findings might meet the request for proper treatment of catatonia in schizophrenia with ECT or rTMS.

## Future Directions on Treatment

Lorazepam is the first choice for treating catatonia, and benzodiazepines are the first-line treatment option. Eighty percent of catatonia is relieved by benzodiazepines or barbiturates, while ECT will be adopted for those patients who fail (Appiani and Castro, 2018). The retrospective studies reported that it is effective in 80 to 100% of all forms of catatonia, including a total of 171 patients (Dutt *et al*., 2011; Raveendranathan *et al*., 2012; Unal *et al*., 2013). It is considered the most effective intervention for catatonia (Ungvari *et al*., 2018). Unal *et al*. (2013) found that catatonia recovered through combined treatment of benzodiazepine and ECT. When drug intervention fails and ECT has safety problems, rTMS is helpful for the acute and maintenance treatment of catatonia in schizophrenia. It is a non-invasive stimulation technique with no adverse effects on cognition compared with ECT (Stip *et al*., 2018). However, due to small-sized studies, there is a lack of solid evidence to support clinically significant results and the application of rTMS in treating schizophrenia with catatonia is limited. In a case report (Stip *et al*., 2017), catatonic symptoms decreased significantly after receiving rTMS targeting bilateral DLPFC. rTMS may be an effective and safe treatment for catatonia. In conclusion, rTMS can be used in patients who are drug non-responsive or contraindicated to ECT. However, more clinical studies are needed to evaluate the suitability of rTMS for the treatment of catatonia. In the future, it may be possible to help the development of precision psychiatry through the mechanism of computational models. The Bush–Francis Catatonia Rating Scale can be used as a measure of symptoms and then a model of the mechanism explaining how rTMS relieves the symptoms of catatonia can be developed.

Our review shows that catatonia patients exhibit decreased connectivity in a network encompassing cortico-striatal-cerebellar pathway, such as decreased structural connectivity between the bilateral medial orbitofrontal cortex and striatum. Catatonia may involve changes in the myelination of corticostriatal connections. In addition, the increase in the severity of catatonia may be associated with high perfusion of SMA. Multiple brain structures differ in patients with catatonia compared to patients without catatonia. These findings suggest that the affected functional and anatomical circuitry of the corticostriatal system is part of the neurobiology of catatonia. Based on this evidence, an alternative approach is a stimulation targeting the affected corticostriatal network in schizophrenia with catatonia revealed by MRI. Although most rTMS studies currently target the DLPFC for the modulation of catatonia, future studies may target the cortico-striatal-cerebellar pathway to explore new targets for treating catatonia. However, the specificity of this pathway needs to be further explored.

## Conclusions

Neurobiological underpinnings contribute to the manifestation of catatonia remains to be determined. Nevertheless, rTMS may serve as an effective and safe treatment for catatonia. The cortico-striatal-cerebellar pathway may be served as a target of rTMS for catatonia.

## Supplementary Material

kkad019_Supplemental_File
