# Supplementary figures and images for: Affected cortico-striatal-cerebellar network in schizophrenia with catatonia revealed by magnetic resonance imaging: indications for electroconvulsive therapy and repetitive transcranial magnetic stimulation
Source: Psychoradiology. 2023 Oct 19;3:kkad019. doi: 10.1093/psyrad/kkad019 (PMC10917379; doi:10.1093/psyrad/kkad019)

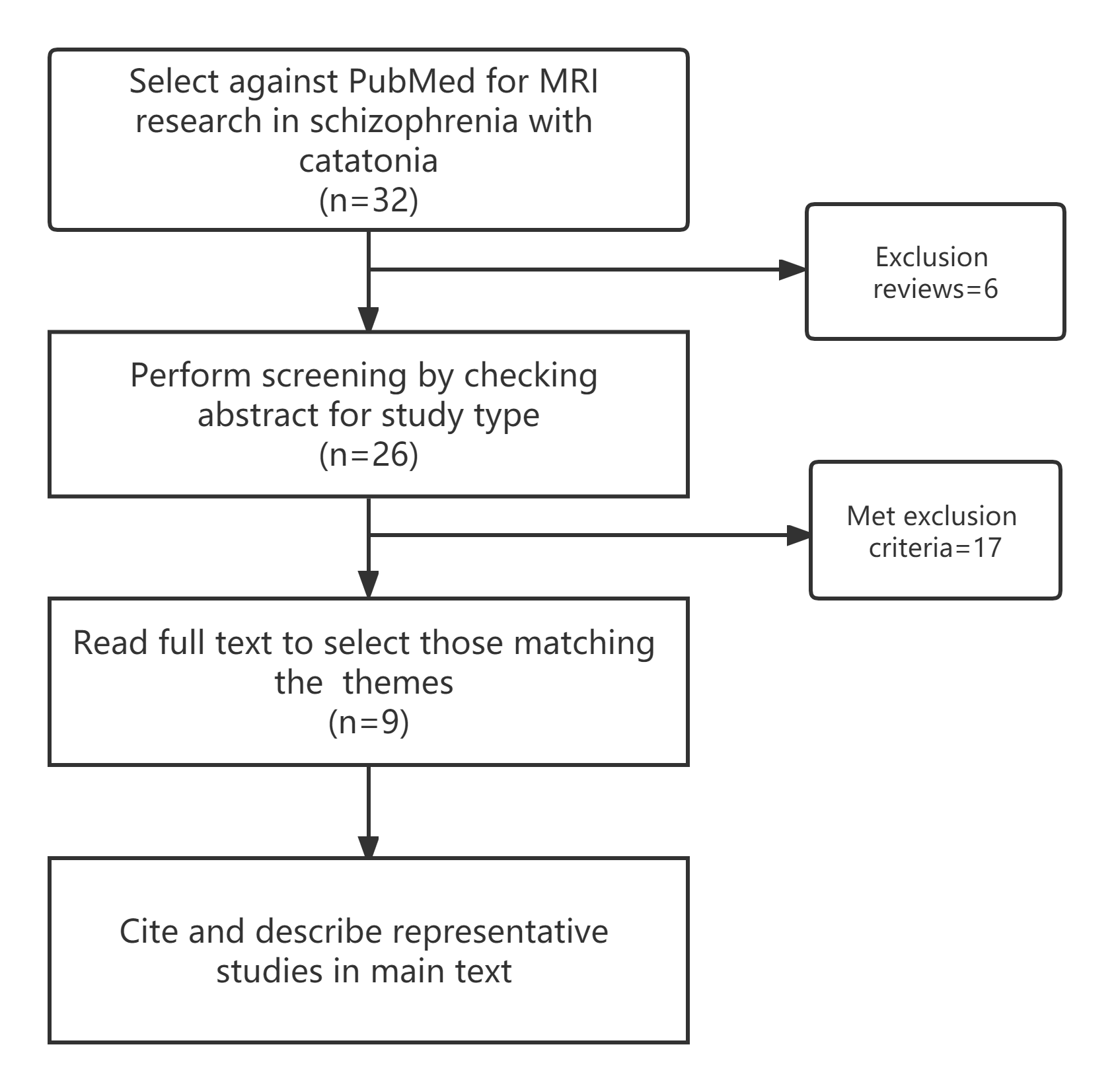

Supplement: kkad019_Supplemental_File [file kkad019_Supplemental_File.zip › Figure_1.tif]
